# Can we measure catalyst efficiency in asymmetric chemical reactions? A theoretical approach

**DOI:** 10.3762/bjoc.5.67

**Published:** 2009-11-19

**Authors:** Shaimaa El-Fayyoumy, Matthew H Todd, Christopher J Richards

**Affiliations:** 1 School of Biological and Chemical Sciences, Queen Mary, University of London, Mile End Road, London, E1 4NS, UK; 2 School of Chemistry, University of Sydney, NSW 2006, Australia; 3 School of Chemical Sciences and Pharmacy, University of East Anglia, Norwich, NR4 7TJ, UK

**Keywords:** asymmetric, catalysis, enzymes, organocatalysis, transition metal complexes

## Abstract

Small molecule asymmetric catalysts are often described as being “good” or “bad” but to date there has been no way of comparing catalyst efficiency quantitatively. We define a simple formula, Asymmetric Catalyst Efficiency (ACE), that allows for such a comparison. We propose that a catalyst is more efficient if fewer atoms are utilised to give a product in a required enantiomeric excess. We illustrate this concept by analysing several well-known asymmetric catalytic chemical reactions carried out in academic laboratories, and compare small molecule catalysts with enzymes. We conclude that ACE is a useful descriptor for the comparison of diverse catalytic systems. It is also noteworthy that, despite the relatively short period of investigation into small molecule catalysts, they are competitive with enzymes with regards to this measure of catalytic efficiency.

## Introduction

The preferential formation of one enantiomer of a molecule via asymmetric catalysis remains one of the most challenging and exciting areas of academic and industrial research in chemistry [1]. Enormous progress has been made in recent years, most notably in homogenous transition metal catalysis, organocatalysis and enzyme-catalysed reactions. It is therefore surprising that there is no generally accepted measure for the effectiveness of a catalytic reaction. That is to say when we anecdotally refer to a “good“ or “bad“ reaction, there is no system for comparing those reactions with each other. Well-known examples of asymmetric catalysis such as the Sharpless asymmetric dihydroxylation, the Corey oxazaborolidine ketone reduction or the proline-catalysed aldol reaction are almost universally deemed ‘good’ in some respect. Enzymes are regarded as highly effective asymmetric catalysts, but is this on the grounds of the difficulty of the transformations they catalyse rather than their practical utility? Are these value judgements fair, and is there a way we might make quantitative comparisons that summarise the diverse features of catalytic, asymmetric reactions?

In this paper we focus on a definition of catalyst efficiency that takes into account the number of atoms involved in effecting the relevant reaction. We evaluate the proposed formula for several well-known catalytic systems that are widely used in laboratories around the world. Our focus here is less on the industrial use of small molecule catalysts, since there are other very specific requirements for the use of catalysts on a manufacturing scale; ours is a more academic consideration of what ‘efficiency’ means when applied to an asymmetric catalyst.

## Discussion

### Definition of Asymmetric Catalyst Efficiency (ACE)

The enantiomeric excess (ee) of a product and the yield of the corresponding reaction are crucial factors in defining “success”, and essentially describe the amount of major enantiomer produced. Clearly also the amount of catalyst required for a given reaction is important, and hence a low loading (mol %) value is advantageous. Are there further factors that might be informative?

**Figure 1:**
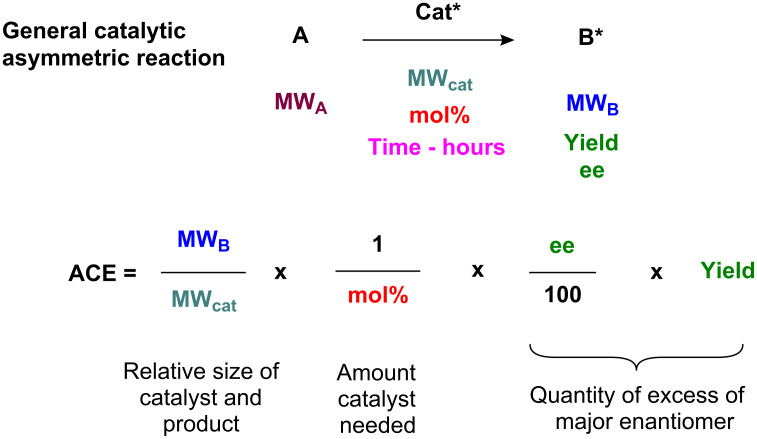
Definition of Asymmetric Catalyst Efficiency (ACE).

We propose that, other things being equal, *a ligand of low molecular mass able to induce asymmetry in a given substrate is more efficient that one of high molecular mass*: the catalyst requires fewer atoms to achieve a relative stabilisation of the transition state. In a parallel with Trost’s assessment of organic reaction “atom economy,” a lower molecular weight catalyst is more atom-efficient [2]. The ratio of the molecular weight of the product to the molecular weight of the catalyst may be used for a calculation of catalyst effectiveness. We thus propose the formula shown in Figure 1 for Asymmetric Catalyst Efficiency (ACE) where all these factors are included. The formula is straightforward in that the relevant values are almost always known for any given catalytic, asymmetric reaction, and yield, ee and mol % are used in their standard forms (i.e. as percentages).

Values for ACE may be obtained for any given catalytic system, and several well-known reactions are shown in Table 1. The primary criterion used for selecting these examples is the commercial availability of the catalysts. We have endeavoured to cover representative reduction, oxidation and C–C bond forming reactions that have been developed in academic laboratories. The values of ACE vary by up to five orders of magnitude. The most efficient small molecule catalyst is that used in a hydrogenation reaction, which tallies well with this method’s extensive industrial usage. An industrial example is shown in entry 4. This is interesting since it illustrates that a high enantiomeric excess need not be the only criterion by which a catalyst is judged: this asymmetric hydrogenation, which gives a product of 79% ee, is employed in the industrial multi-tonne synthesis of (*S*)-metolachlor [3]. An instructive comparison may be made between an antibody capable of catalysing an intramolecular, asymmetric aldol reaction and proline, capable of catalysing the same reaction (the Hajos–Parrish–Eder–Sauer–Wiechert reaction – entries 8 and 9). Proline performs slightly better in this reaction, despite being used at a loading of 48 mol % (!) in one of the original reports, and this is partly due to the very large molecular weight of the antibody.

**Table 1:** Calculation of ACE for various catalytic asymmetric transformations.

Entry [Ref.]	Reaction	Catalyst/Catalyst components	Yield (ee) [%]	**ACE**	Catalyst cost ^a^ [Eur]	Cost of 1 mmol of excess enantiomer of **B** [Eur]^b^	Turnover (h^−1^)	ACES (h^−1^)

1 [6]	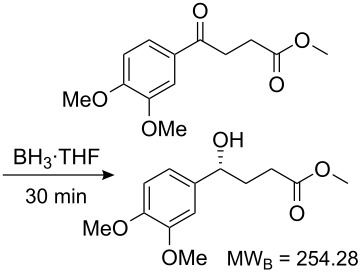	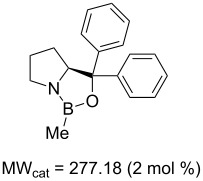	98 (95)	**42.7**	52.20	0.31	98	85.4
2 [7]	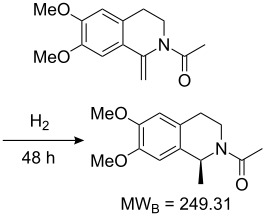	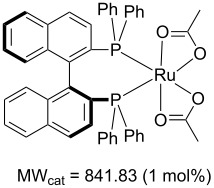	100 (96)	**28.4**	144.40	1.27	2.1	0.59
3 [8]	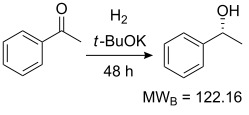	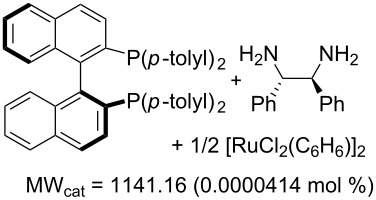	100 (80)	**206858**	119.50	0.00007	50322	4310
4 [3]	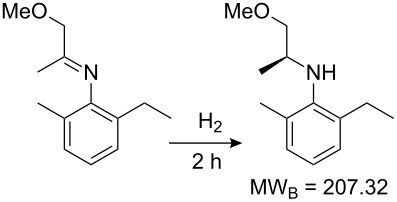	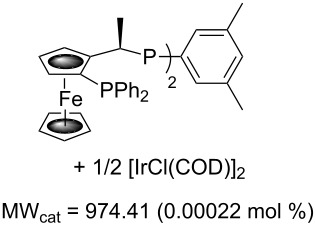	99.6 (79)	**76096**	381.71	0.001	226364	38048
5 [9]	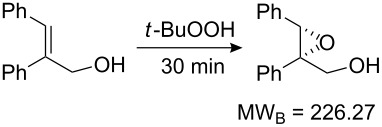	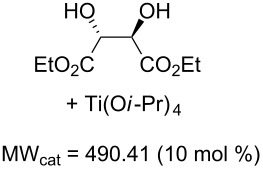	87 (95)	**3.81**	0.21	0.012	17	7.62
6 [10]	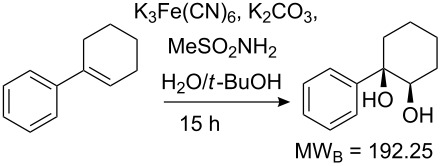	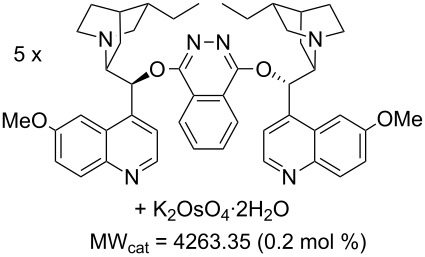	89 (99)	**19.9**	65.81	0.64	30	1.33
7 [11]	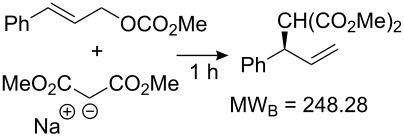	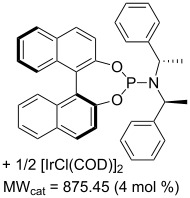	88 (96)	**5.99**	334.32	13.9	22	5.99
8 [12]^c^, [13]	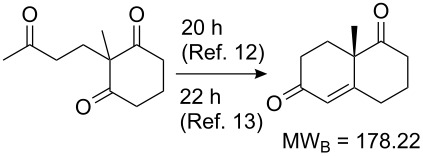	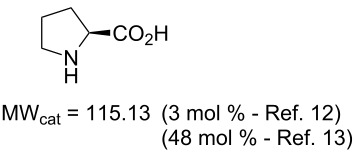	94 (95) 86 (84)	**46.1** **2.33**	0.76 0.76	0.0029 0.058	1.6 0.08	2.31 0.11
9 [14]	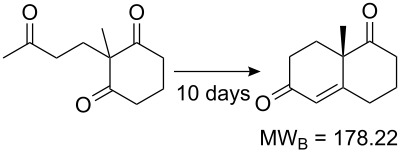	Antibody 38C2 MW_cat_ ~150000 [15] (0.114 mol %)	94 (95)	**0.93**	23650	4532	3.4	0.004^d^
10 [16]	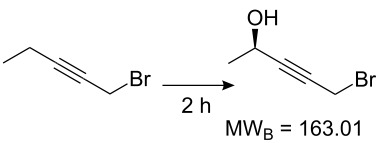	Chloroperoxidase MW_cat_ = 42000 [17] (0.11 mol %)	65 (94)	**2.16**	^e^	0.161	295	1.08
11 [18]	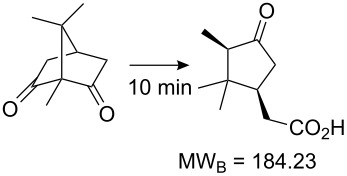	6-Oxocamphor hydrolase MW_cat_ = 83000 [19] (0.00068 mol %)	86 (95)	**267**	n/a	n/a	758823	1602
12 [20]	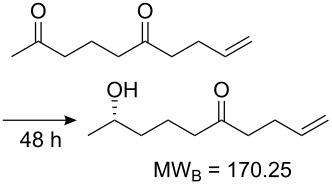	*Thermoanaerobacter brockii* alcohol dehydrogenase MW_cat_ = 40000 [21] (0.0035 mol %)	60 (99)	**72.3**	^f^	125	357	1.51

^a^Catalogue prices (2009) of 1 g of the less expensive enantiomer, if the two differ. ^b^The cost of 1 mmol of the excess of the major enantiomer given by MW_B_/1000 × catalyst cost[1g]/ACE. ^c^Initial aldol reaction (20 h) followed by a separate dehydration reaction. ^d^Catalytic antibodies are known for related reactions that are faster [22], 38C2 was chosen as a comparison with proline since the reaction catalysed here is the same, the mass of catalyst is known for this reaction, and the antibody is commercially available. ^e^1 mL, >10000U/mL, Eur 189.5. ^f^Cost based on alcohol dehydrogenase from *T. brockii*, 100 U, Eur 144.5.

It can be challenging to apply the ACE formula to enzyme-catalysed reactions because frequently it is not known how much enzyme is being employed in a given transformation. Cell-free extracts are commonly prepared and assayed for their ability to convert a certain amount of substrate in a certain amount of time, without quantification of the mass of enzyme in that preparation. We have chosen an oxidation, a reduction and a hydrolytic desymmetrisation where the quantity of enzyme is specified, and the ACE values for these reactions are shown (Table 1, entries 10–12). High ACE values may be obtained, but the very large molecular weight of the enzyme required to effect the relevant transformations reduces their competitiveness with respect to small molecule catalysts.

Of particular note with the proposed formula is that multiplying ACE by the amount (in grams) of catalyst employed gives the amount (in grams) of the excess of the major enantiomer produced by the reaction. Thus use of ACE permits a simple calculation of the cost of the catalysts for the various transformations. These are included in Table 1 together with a value of the normalised catalyst cost for the synthesis of 1 mmol of the excess of the major enantiomer of product. It can be seen that small molecule catalysts perform exceptionally well from this economic perspective, with the asymmetric hydrogenations again leading the field.

A distinction is obviously required between what is added to a reaction mixture as the catalyst, and what is the actual catalytically active species. In most cases these will differ. If we are to quantify the real ability of a catalyst to carry out an enantioselective transformation, we should use MW_cat_ for the species formed in situ, if it is known. For this one should consider the sum total of the transition state components minus those atoms ending up in the product. In many cases, however, the exact identity of the active catalyst and/or the identity of the transition state of the enantioselective step are not known. To maintain the correspondence between (amount of catalyst added × ACE) and quantity of the excess of the major enantiomer formed, it is useful to use, as MW_cat_, the molecular weight of the material added to the reaction mixture, ignoring other additives, or the nature of the transition state for the reaction. This is practically useful as a catalyst is typically used “as is”.

There exist other descriptors of catalytic ability. The turnover number (TON) is typically taken to be the number of moles of product produced per mole of catalyst, and the turnover frequency (TOF) equals the TON per unit time of reaction. The Productivity Number of an enzyme is the amount of reaction product divided by the dry weight of catalyst used and the reaction time [4]. The SI unit for efficiency is the katal (kat): a catalyst converting one mole of substrate in one second has an activity of 1 kat [5]. This unit has not yet been widely embraced (possibly due to the large size of each kat), and certainly is not used for small molecule catalysts. For enzymes, the non-standard Unit (U) is often used, where a catalyst preparation capable of turning over one μmol per minute has an activity of 1 U. The theoretical effectiveness of enzyme action is discussed by reference to *k*_cat_/*K*_m_, the Michaelis–Menten parameters, and *V*_max_. These values are for maximal velocity under ideal conditions. It is interesting to note how infrequently the equivalent values are known for small molecules: maximal rate is usually not known for small molecule catalysts, and mol % and yield are typically not of primary concern to enzymologists. As an assessment of efficiency, *k*_cat_ or *V*_max_ fall short since they do not take into account other factors of preparative importance, such as the catalyst’s molecular weight. The ACE value embodies a more practical assessment of catalyst efficiency centred on *preparative usefulness*, where catalyst loading, molecular weight, and by extension, cost, can be considered. Many catalytic, asymmetric reactions that are of preparative interest to chemists have time frames ranging from minutes to hours, and where the ability of the catalyst to turn over substrate is reflected in the amount of catalyst that needs to be added. It is instructive to compare ACE with the ‘turnover’ based on the time taken for the reaction to finish, i.e. the average number of substrate molecules processed by the catalyst during the reaction per hour (yield/(mol % × time)) which in many cases will be unoptimised. These values are shown in Table 1. We define the Asymmetric Catalyst Efficiency Speed (ACES) as ACE/t and these values are also shown in Table 1. While turnover is based purely on yield, mol % and time, ACES additionally includes relative molecular weights of catalyst and product, as well as ee.

Factors other than those considered here will be important in explaining the adoption of a catalyst, or a particular chemical route, by industry. Cost of starting materials, and the hazards involved in a process are key considerations from a manufacturing perspective. Aqueous or air compatibility and recyclability are two features of a catalyst that may offset a low ACE, for example. Other factors include reaction temperature and the ease of removal of a catalyst, especially those based upon toxic heavy metals such as palladium. A catalyst may be particularly useful if it accelerates a transformation on a challenging starting material, or a reaction for which there currently exist few alternatives. The Sharpless Asymmetric Epoxidation, for example, suffers from a low ACE. From an academic viewpoint, this transformation is nevertheless important because of the breakthrough nature of this relatively low cost reaction. It has few industrial uses perhaps because of issues of safety, but these are difficult to quantify for our purposes. Nevertheless ACE combines the key parameters describing the practical usefulness of a catalyst for a laboratory not involved in bulk manufacture.

While it is our aim to define a simple formula allowing a comparison of reactions commonly described in the academic chemical literature, the main emphasis of this commentary is on a consideration of atom economy applied to an asymmetric reaction. Thus a small ligand with few atoms able to catalyse a reaction as effectively as a larger ligand requiring more atoms to effect the same transformation is more efficient. Naturally, the exact ACE value for any given reaction depends on the product molecular mass, since we are using this in the ACE formula as a comparison with the catalyst molecular mass. ACE is defined for a given chemical reaction, and will vary across a series of substrates, which is perfectly reasonable since the effectiveness of a transformation is also influenced by variation in starting material. *ACE thus describes a catalyst applied to a specific chemical reaction*.

## Conclusion

We have defined asymmetric catalyst efficiency (ACE) by a formula that includes, in addition to the ee and yield of the product, the amount of catalyst employed and the relative size of the catalyst to the product. Comparison of the ACE values calculated for a series of representative reactions utilising commercially available catalysts highlights the extraordinary efficiency of ligated transition metal catalysts in asymmetric hydrogenation. An analysis of the unit cost of each catalyst reveals these to span a very wide range, with only the amino acid proline comparing favourably with the hydrogenation systems. The ACE formula permits a comparison between small molecule catalysts and enzymes. The results, covering a mere thirty years of research, are testament to the extraordinary progress that has been made in small-molecule asymmetric catalysis.

## References

[1] Halpern J, Trost B M (2004). Proc Natl Acad Sci U S A.

[2] Trost B M (1991). Science.

[3] Blaser H-U (2002). Adv Synth Catal.

[4] Simon H, Bader J, Günther H, Neumann S, Thanos J (1985). Angew Chem, Int Ed Engl.

[5] Dybkær R (2001). Pure Appl Chem.

[6] Corey E J, Bakshi R K, Shibata S (1987). J Am Chem Soc.

[7] Noyori R, Ohta M, Hsiao Y, Kitamura M, Ohta T, Takaya H (1986). J Am Chem Soc.

[8] Doucet H, Ohkuma T, Murata K, Yokozawa T, Kozawa M, Katayama E, England A F, Ikariya T, Noyori R (1998). Angew Chem, Int Ed Engl.

[9] Katsuki T, Sharpless K B (1980). J Am Chem Soc.

[10] Sharpless K B, Amberg W, Bennani Y L, Crispino G A, Hartung J, Jeong K-S, Kwong H-L, Morikawa K, Wang Z-M, Xu D (1992). J Org Chem.

[11] Streiff S, Welter C, Schelwies M, Lipowsky G, Miller N, Helmchen G (2005). Chem Commun.

[12] Hajos Z G, Parrish D R (1974). J Org Chem.

[13] Eder U, Sauer G, Wiechert R (1971). Angew Chem, Int Ed Engl.

[14] Zhong G, Hoffmann T, Lerner R A, Danishefsky S, Barbas C F (1997). J Am Chem Soc.

[R15] 15Aldrich Catalogue and personal communication from C. F. Barbas III (to MHT).

[16] Hu S, Hager L P (1999). J Am Chem Soc.

[17] Morris D R, Hager L P (1966). J Biol Chem.

[18] Grogan G, Graf J, Jones A, Parsons S, Turner N J, Flitsch S L (2001). Angew Chem, Int Ed.

[19] Grogan G, Roberts G A, Bougioukou D, Turner N J, Flitsch S L (2001). J Biol Chem.

[20] Keinan E, Sinha S C, Sinha-Bagchi A (1991). J Chem Soc, Perkin Trans 1.

[21] Lamed R J, Zeikus J G (1981). Biochem J.

[22] Zhong G, Lerner R A, Barbas C F (1999). Angew Chem, Int Ed.

